# Clinical procedure for intraocular electrochemical lysis during endoresection

**DOI:** 10.3332/ecancer.2013.326

**Published:** 2013-06-20

**Authors:** YA Belyy, AV Tereshchenko, AV Shatskih

**Affiliations:** 1 Academician Fedorov Interdisciplinary Scientific and Technological Complex ‘Eye Microsurgery,’ Kaluga Branch, Ministry of Public Health and Social Development of the Russian Federation, Kaluga, Russia; 2 Academician Fedorov Interdisciplinary Scientific and Technological Complex ‘Eye Microsurgery,’ Ministry of Public Health and Social Development of the Russian Federation, Moscow, Russia

**Keywords:** large choroidal melanoma, intraocular electrochemical lysis, endoresection

## Abstract

Optimal clinical approaches to the treatment of large intraocular lesions located in the posterior pole of the eye remain controversial. In our opinion, the introduction of innovative techniques into the clinical practice of vitreoretinal surgery professionals as well as the implementation of new techniques designed to destroy tumour tissue will make endoresection one of the most promising alternative approaches to choroidal melanoma management, providing an encouraging high-potential eye-saving treatment option in the future. However, in view of a small number of treated cases and a short follow-up period, further research studies are required to evaluate the effectiveness of the electrochemical lysis procedure during endoresection.

## Introduction

Choroidal melanoma (CM) is characterised by an extremely poor prognosis, endangering both the visual functions in patients and their lives, due to a high risk of metastatic disease (3%–16%) [3, 12, 14]. In line with the modern approach to eye cancer management, the most preferable options for CM treatment are those meeting the requirement for maximally radical measures to be taken to eradicate the neoplasm while inducing minimal damage to the adjacent tissues. The applicability of organ-saving techniques in CM treatment generally depends on the tumour’s size and its specific location. Large tumours are usually managed by eye enucleation.

The range of organ-saving techniques available for the treatment of CMs located in the posterior pole of the eye is rather limited: transpupillary thermotherapy (TTT), transscleral thermotherapy, photodynamic therapy (PDT), radiation therapy, and brachytherapy.

Attempts to modify commonly accepted parameters of the techniques (TTT, PDT, brachytherapy) used in large CM treatment, with the purpose of increasing their therapeutic effect, generally result in a higher number of complications, both intraoperative and postoperative [2, 6, 8, 9]. It should also be noted that, following the application of these techniques, individually or in combination, large volumes of semi- or totally necrotised cell residue remain in the ocular cavity for a long period (reabsorption period), affecting adjacent eye tissues.

With this in mind, we decided to take a new look at endoresection as an alternative organ-saving technique of surgical treatment of large CMs.

This technique, as it has been described in a number of studies [5, 10, 11, 13, 15], has certain obvious disadvantages. They include a high risk of cancer cell migration, possible iatrogenic retinal damage, haemorrhage, technical problems associated with the procedure of tumour removal, and risk of further tumour growth. Therefore, the endoresection process has to be modified and upgraded.

## Objective

The objective is to develop an innovative combined approach to endoresection of large intraocular tumours localised in the posterior eye pole, involving intraocular electrochemical lysis (ECL) at the stage of tumour destruction.

## Materials and methods

Three patients (three eyes) with large CMs (T_3_N_0_M_0_) (tumour thickness of 8–10 mm, basal diameter of 13–15 mm, juxtapapillary localisation) received endoresection coupled with intraocular ECL. The average age of the patients was 55.4 years.

Ophthalmoscopic examination showed that the lesion colour was grey; visual acuity testing revealed abnormal light reflex in all patients.

The findings obtained with grey-scale B-scan ultrasonography revealed a hyperechogenic tumour with a clearly outlined irregular configuration. In two eyes, secondary exudative retinal detachment was found. Colour Doppler imaging tests showed a hypovascular-type tumour in all patients.

**Figure 1:**
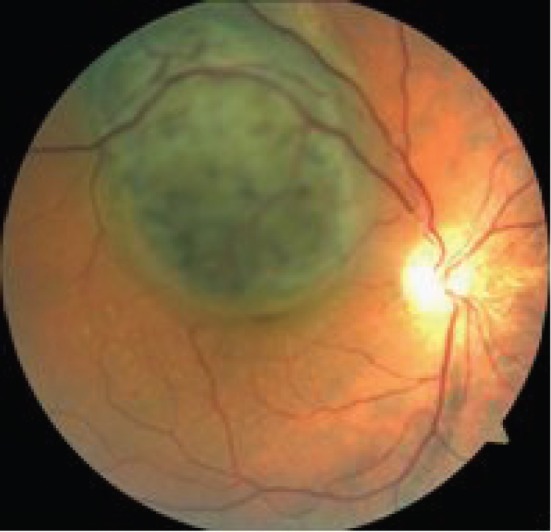
A large CM located in the posterior eye pole.

Based on the results of complex ophthalmological examination, all patients were diagnosed with CM OD (Figure 1).

Considering the size and location of the malignant lesions, endoresection with intraocular ECL was recommended to the patients, and their informed consent was subsequently obtained.

**Figure 2:**
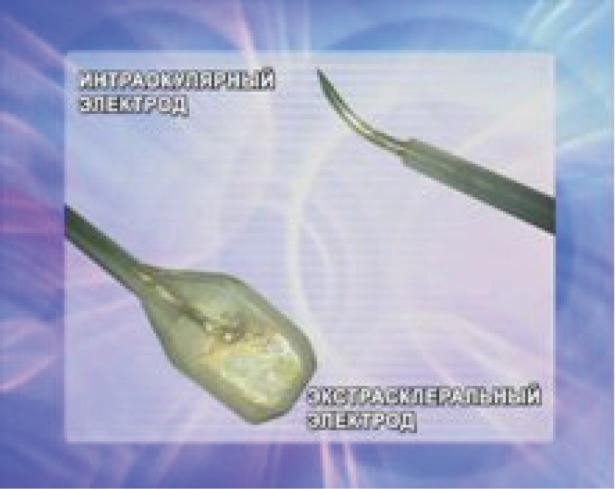
Extrascleral and intrastromal electrodes for the ECL procedure.

ECL was performed using an ECU 300 device (Söring, Germany) with an electric charge of 20–25 coulombs. During the ECL procedure, an innovative technique of combined positioning of two electrodes was used, with one surface (extrascleral) electrode serving as the anode, and the other (intraocular) electrode serving as the cathode. The extrascleral electrode was made in the form of a platinum wire mesh (wire diameter of 0.05 mm and mesh size of 0.1 mm). The electrode diameter was 3 mm. The lower surface of the electrode was embedded into an insulating material, while its upper surface remained exposed. The electrode had a handle in the form of an angulated spatula that allowed extrascleral manipulations to be performed to guide the electrode into the area of the tumour basal projection, suitable for further moving it along the scleral surface within the tumour outline. The intraocular electrode was made in the form of a 23 G platinum needle with a curved intratumoural tip. With the exception of its intratumoural section, the electrode was coated with biologically inactive dielectric material (Figure 2).

Before starting endoresection, phacoemulsification was done through a scleral incision. Following this, a 23 G three-port vitrectomy was performed by removing the posterior hyaloid membrane, and restrictive endolaser coagulation (λ = 532 nm) was done in three coagulate rows around the tumour at a distance of 2–3 mm from the visible edge. It was followed by retinectomy along the coagulated border, the retina was separated off, and the tumour was fully exposed. Laser coagulation of the choroid (λ = 810 nm) in continuous circular-wave mode was performed at 1 mm from the retinectomy borders. The next step included air–fluid exchange. Subsequently, an endoilluminating device (27 or 29 G) was positioned transsclerally at 4.4 mm from the limbus.

The incision of the conjunctive and Tenon’s membrane was made to insert and position the electrode (anode) extrasclerally within the most suitable tumour meridian at 5–6 mm from the limbus. Using the spatula, a tunnel was created and, with the help of the handle, the electrode was guided through it to the area of the tumour projection on the sclera, so that a close contact between the electrode and the sclera could be maintained. To control the proper electrode positioning, sclerocompression was used. An assistant surgeon was holding the handle tightly to keep the electrode in place.

**Figure 3:**
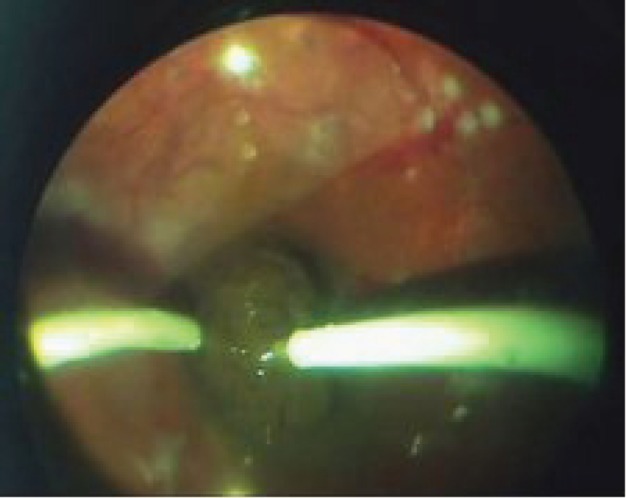
Intrastromal electrode (cathode) insertion in the tumour tissue.

A pars plana sclerotomy was performed at 3.5 mm from the limbus in the quadrant that allowed the best possible access to the tumour, and the intraocular electrode (cathode) was inserted intravitreally (Figure 3). The intratumoural section of the electrode was guided under direct visual control to the tumour site, with subsequent implantation into the neoplasm parallel to the scleral plane and at 2–3 mm from the tumour apex.

**Figure 4:**
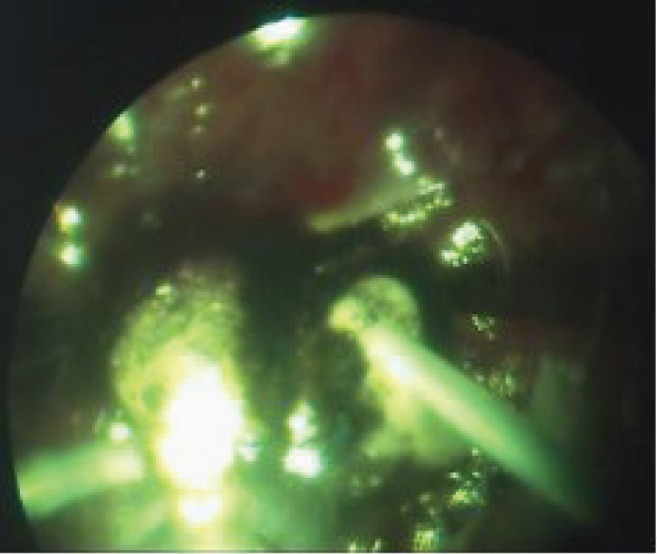
The ECL procedure.

**Figure 5:**
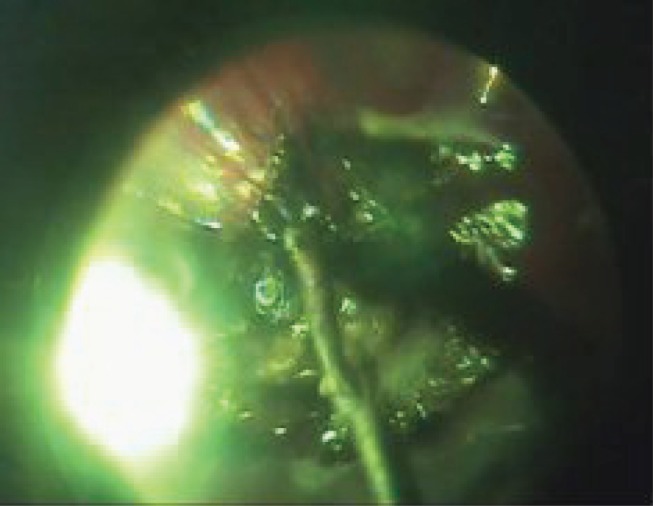
Destructed tumour tissue removal with a vitreous cutter.

Once the extrascleral and intraocular electrodes were in place, the ECL procedure was performed at the amperage of 20–25 mA within a period, which was required to decompose the tumour around the cathode; the decomposition process was accompanied by the formation of liquid cell debris that was removed during the ECL procedure using a vitreous cutter (Figures 4 and 5). As the electrochemical decomposition of the tumour progressed and cellular debris was removed, the intraocular electrode was moved closer to the tumour base, with care being taken to maintain the electrode position parallel to the scleral plane. Upon reaching the tumour segment adjacent to the sclera, the ECL procedure was discontinued, the assistant surgeon changed the position of the extrascleral electrode using sclerocompression to control proper electrode placement, the electrode was fixed in place with the help of the electrode handle, and the ECL procedure was resumed. The extrascleral electrode was moved as many times as necessary to treat the entire area of the tumour basal projection on the sclera.

The effectiveness of the ECL procedure was evaluated by means of bioimpedancemetry—the process of measuring malignant tissue impedance within the area between the electrodes when it was exposed to alternating variable frequency electric current. Multiple changes in lysed tissue impedance were measured using a pilot unit at frequencies of 2 and 10 kHz; to obtain the impedance values, the ECL procedure was interrupted for 1 or 2 s at 10-s intervals. Bioimpedancemetry was performed using the same electrodes as for the ECL procedures. Impedance values (*Z*) were automatically recorded, and the software application was plotting the real-time tissue impedance curve. Stable impedance readings with minimal fluctuations (*Z*) served as a prognostic criterion for tumour necrosis.

Upon completion of the ECL procedure, the electrodes were removed, and decomposed tumour residues were cleaned up using a vitreous cutter, until the bare sclera was exposed within the boundaries of the previously performed choroid circular continuous wave laser coagulation. That was followed by retinectomy and additional endolaser coagulation; the vitreous cavity was filled with silicone oil.

Follow-up observation was done at one week, one and three months, and every six months for the next three years.

Postoperative examination included visometry, tonometry, biomicroscopy, ophthalmoscopy, assessments of tumour recurrence or metastatic disease.

## Results

**Figure 6:**
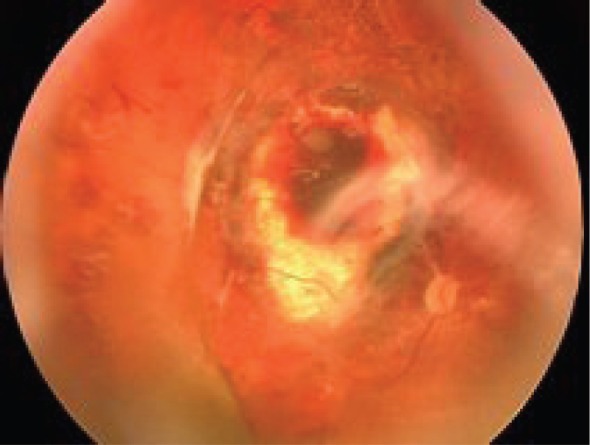
Fundus: one month after endoresection.

In all three cases, the tumour was entirely removed without any complications, and anatomical attachment of the retina was achieved in the course of the surgical procedure. The mean duration of an ECL session including the time required to obtain bioimpedance data was 15 min. As a result of the ECL procedure, the scleral integrity of the eye was retained. Early postoperative complications seen in all patients included moderate subretinal haemorrhages in the scleral bed around the surgical coloboma; the haemorrhages spontaneously resolved within two to four weeks (Figure 6). Elevated intraocular pressure (IOP) was seen in one case and was managed conservatively.

Due to the central tumour location, there were no postoperative changes in visual acuity (abnormal light reflex).

As to long-term postoperative results (1.5–3 years after the surgery), ocular fundus examination in all cases showed the surgical coloboma of the choroid located at the site of the excised intraocular neoplasm, with no signs of pigmentation over the entire scleral bed or around the periphery. Neoplasm recurrence or remote metastasis was not found in any case.

In the late postoperative period, one patient who had previously suffered from 10-mm thick CM was found to have recurrent retinal detachment in the silicone-oil-filled eye due to the rupture over one of the retinopexia edges, which was associated with progressive proliferative vitreoretinopathy. The patient did not receive any repeated surgical treatment.

## Discussion

In our opinion, endoresection of large intraocular tumours with posterior location suggests total tumour removal within the boundaries of the intact tissues, as distinguished from other organ-saving techniques that cause destruction and absorption of necrotised tissue due to therapeutic exposure with subsequent formation of conjunctive chorioretinal scars; these scars can retain viable tumour cells and become a focus for neoplasm recurrence with poor prognosis for repeated treatment options. Endoresection disadvantages include the risk of viable tumour cell dissemination, technical problems associated with the neoplasm removal, haemorrhage, and retinal detachment. Our new endoresection technique has been developed on the basis of the experience and results reported by the authors [10, 11, 13, 15] who used that surgical procedure before.

At present, endoresection is commonly used after irradiation therapy, brachytherapy, TTT, or PDT. In our opinion, however, this approach is associated with a number of technical problems due to the scar tissue formation, making it difficult to remove the tissue by a vitreous cutter and requiring the use of additional vitreoretinal instruments. As a result, the surgical procedure takes more time, and the risk of intraoperational complications is increased. Therefore, endoresection should be coupled with intraoperative procedures, inducing destruction and pathological changes in the tumour structure and then allowing easy lesion removal.

ECL provides an appealing example of using this approach for cancer treatment. The approach is based on destructive chemical reactions induced by direct electric current flowing between two electrodes placed inside the tumour, resulting in tumour tissue devitalisation due to electrolysis.

A growing interest in using ECL is mostly related to distinct clinical effect which, in addition to its relative affordability and availability, is shown in a great number of medical publications [1, 4, 7, 16–18].

An incentivising factor that has encouraged us to develop and introduce into clinical practice the technique based on ECL application for the treatment of intraocular malignant lesions was the fact that neither such a technique nor any clinical data on its effectiveness were available.

Our previous experiments involving ECL treatment of enucleated eyes with intraocular tumours demonstrated that intratumoural implantation of the electrodes (the anode and the cathode) resulted in acid-induced necrosis around the anode electrode, with subsequent formation of thickened necrotic tissue, which was difficult to remove by a vitreous cutter. Tumour tissue decomposition around the cathode was accompanied by the formation of liquid cell debris (alkaline-induced necrosis) that allowed easy lesion removal without any technical problems. This finding formed a basis for the development of an innovative combined technique that involved the use of intraocular ECL, with surface and intrastromal electrodes used during endoresection.

An innovative method of positioning the electrodes during ECL allows the entire surgical procedure to be performed without any need to modify a conventional three-port endoresection technique.

During the ECL procedure, liquid cell debris with totally destructed cells was formed around the intraocular cathode electrode; the resultant effect made it possible to easily remove the entire tumour mass using a vitreous cutter tip (23 G). The extrascelral position of the anode electrode, allowing the electrode relocation and sclerocompression, provided an opportunity to treat the entire scleral surface within the outline of the neoplasm projection area and, therefore, to reduce the risk of viable tumour cell survival.

Intraocular water replacement with air and long-lasting silicone oil tamponade of the vitreous cavity during endoresection significantly reduced tumour cell dissemination and metastatic processes.

During the ECL procedure with combined electrode positioning, we used experimentally derived electric charge parameters—20–25 coulombs. These parameters provided irreversible necrotic changes, did not lead to active bubble formation that complicated the visualisation of the ongoing processes, and were painless for the patients.

To evaluate the effectiveness of the procedure, we used the method of impedancemetry for ECL exposed tissues, which allowed the dynamics of malignant tissue impedance changes to be assessed and the impedance termination moment to be determined.

In the course of the ECL procedure, the intraocular electrode was moved along the area within the tumour borders, causing total lesion destruction. The resistance drop (*Z*) and stable impedance readings with minimal fluctuations over time in the given portion of the tumour served as a prognostic criterion for the tumour necrosis.

The proposed ECL technique enabled us to reach haemostasis in patients undergoing the procedure: no haemorrhages occurred during the tumour resection. It is associated with biological tissue response to electromagnetic field, which is characterised by blocking the vascular bed. Microthrombosis of the tumour basal vascular bed was seen around the extrascleral anode, while the capillary thrombosis around the cathode was due to the electro-osmotic flow.

The application of laser irradiation (with a wavelength of 810 nm) to perform continuous-wave coagulation of the choroid around the intraocular neoplasm was also one of the ways to control haemorrhage and to facilitate the tumour endoresection, following the ECL procedure, thus localising the tissue being incised.

Therefore, the modification, refinement and customising of the intraocular ECL procedure as a new eye cancer management modality help to achieve total necrosis and structural changes of intraocular lesions during the surgery and thus increase endoresection efficiency; while the development of objective and real-time assessment tools to determine the extent of pathologic changes within the tumour structures— measuring active and reactive resistivity of malignant tissue (bioimpedancemetry)—provides the basis for necessary control and monitoring during the ECL procedure.

## Conclusion

The treatment of large CM localized in the posterior pole of the eye has been and remains controversial. In our opinion, the introduction of innovative techniques into vitreoretinal surgery and the development of intraocular procedures designed to destroy tumour tissue make endoresection a promising and encouraging organ-saving treatment option.
